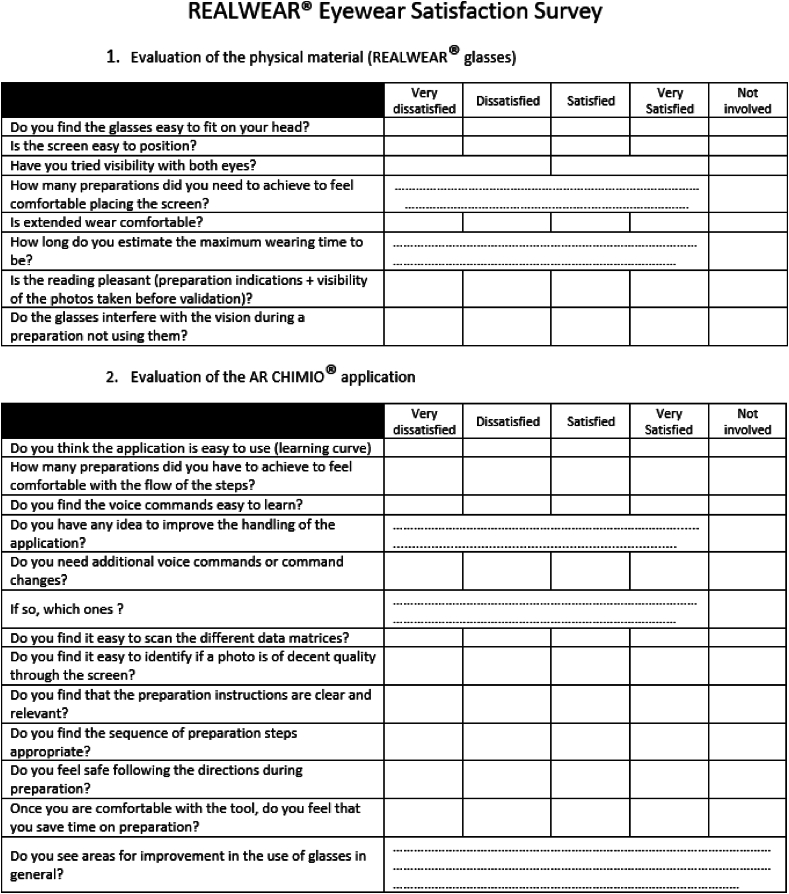# A prospective, single-centre study of the feasibility of the use of augmented reality for improving the safety and traceability of injectable investigational cancer drug compounding

**DOI:** 10.1016/j.heliyon.2024.e32683

**Published:** 2024-06-10

**Authors:** Arthur Lecoutre, Michele Vasseur, Justin Courtin, Slim Hammadi, Bertrand Decaudin, Odou Pascal

**Affiliations:** a CHU Lille, Institut de Pharmacie, F-59000, Lille, France; b Univ. Lille, CHU Lille, ULR7365 – GRITA – Groupe de Recherches sur les Formes Injectables et les Technologies Associées, F-59000, Lille, France; c Univ. Lille, CNRS, Centrale Lille, UMR 9189 CRIStAL, F-59000, Lille, France

**Keywords:** Chemotherapy, Clinical trials, Preparation, Quality control, Augmented reality, User satisfaction

## Abstract

The compounding of injectable cancer drugs for clinical trials often requires specific procedures, with limited access to the starting materials and especially the active compound. These characteristics prevent the application of qualitative or quantitative analyses and quality control techniques. Hence, for some very complex compounding operations, double visual inspection is considered to be less reliable, more time-consuming and more human-resource-intensive than other methods. The compounding team at Lille University Hospital (Lille, France) has equipped one of its preparation areas with a new device: augmented reality (AR) eyewear connected to an oncology drug management system, as a support tool for compounding and quality control. The tool has been tested, adapted and improved within the unit and is now used for investigational drug compounding on a routine basis. In a prospective, single-centre study, we evaluated the feasibility of the implementation of this novel AR approach for the compounding of injectable investigational cancer drugs. During the 6-month study period, 564 clinical trial compounding operations were performed with the AR eyewear. The proportion of poor-quality photos taken with the AR eyewear fell over time, as users became more familiar with the tool. A user satisfaction survey highlighted a very high level of uptake and a wish to broaden the scope of the compounding performed with AR support. The AR eyewear constitutes an innovative, cost-effective tool that increased the level of safety without disrupting the unit's operating procedures. The tool's flexibility enabled its integration into a variety of working environments. The various improvements now being developed should help to further boost the added value of this novel device.

## 1 Introduction

Thanks to the promotion of research and innovation, the number and diversity of therapeutics used in the fight against cancer have increased significantly over recent years. Thus, in October 2019, 48 % of the world's registered clinical trials concerned oncology [1]. The increases in the number of people to be treated and the number of clinical trials have forced healthcare establishments to meet this demand by developing their material and human resources. Hence, the preparation of injectable cancer drug formulations now tends to be concentrated within larger centres with significant production capacities and greater access to innovative technologies and preparation techniques. Injectable cancer drug compounding is a high-risk activity for both the operator and the patient to be treated. A controlled, sterile environment and personal, protective equipment are required. The implementation of rigorous quality control procedures ensures the preparation of the right dose of the right drug for the right patient [2,3]. Good pharmaceutical practice does not impose a particular quality control method; the choice is left to the pharmacist, as long as the chosen method guarantees the preparation's safety and traceability. Many quality control methods can be applied to the preparation of injectable cancer drugs, although each has advantages and disadvantages [4].

The preparation of injectable formulations of investigational cancer drugs sometimes requires particular compounding procedures, with sometimes limited access to raw materials in general and the active compound in particular; this may prevent the development of qualitative or quantitative quality control techniques (e.g. assays and gravimetric analyses). Most quality controls are therefore based on double visual inspection. Moreover, the compounding procedures in clinical trials (and especially Phase I trials) may be complex, with serial dilutions. In fact, the solutions provided by the study sponsor are often much more concentrated than the dose ranges being tested. For very complex compounding, the most frequent “*in process*” quality control method is double visual inspection – generally acknowledged to be the least reliable and most time-consuming, human-resource-intensive approach [[4], [5], [6], [7], [8]].

To address these safety and traceability issues, our hospital has created a computerized circuit for the compounding of injectable cancer drugs by adding an augmented reality (AR) interface (eyewear). The objective is to provide the user with useful information at each step in the preparation process and to enable him/her to take photos during the steps that require double visual inspection [9].

The computerization of the compounding process and the integration of innovative preparation support, quality control and traceability tools is part of our hospital's continuous improvement approach. However, this requires the implementation of qualification procedures, an evaluation of the impact on the quality of the pharmaceutical production, and the users' views. The objective of the present prospective, single-centre study was therefore to assess the feasibility of the implementation of novel AR technology for the compounding of injectable investigational cancer drugs.

## 2 Materials and methods

### 2.1 The study setting

**Fig. 1:**
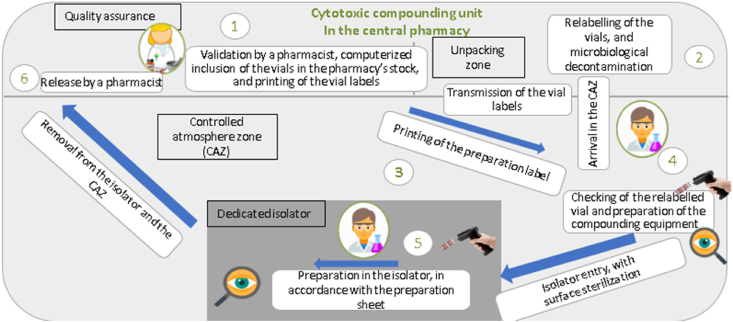
Schematic diagram of the central pharmacy's injectable cancer drug preparation circuit.

The study was conducted at Lille University Hospital (Lille, France). The central pharmacy includes an injectable cancer drug preparation unit with three isolators and six workstations (Sieve France, Villeurbanne, France), a combined UV-Raman spectrometer (QC-Prep®, Icône Services, Sucé-sur-Erdre, France) for quality control, and a RIVA® compounding robot (ARxIUM Inc., Winnipeg, Canada). Lille University Hospital uses the CHIMIO® oncology drug management system (ODMS) from Computer Engineering SARL (Paris, France). The compounding circuit is fully computerized, and operators, raw materials and preparations are identified by barcodes (Fig. 1). The clinical trials preparation circuit comprised six stages. Stage 1 involved validation of the prescription by a pharmacist, inclusion of the vials in the pharmacy's stock, and automatic printing of the Datamatrix labels. In stage 2, the Datamatrix labels were attached to the clinical trial vials, which were then surface-sterilized for entry into the cleanroom. The third stage consisted in printing the preparation label with its unique barcode identifier. In the fourth stage, the vials and labels were checked, and the equipment required for compounding was prepared. Stage 5 consisted of making the preparation in an isolator, with a second operator performing a double visual check at each step. The sixth and final stage was release of the preparation by a pharmacist. In terms of personnel, the unit has three pharmacists, two intern pharmacists, eight pharmacy technicians, and one logistics assistant. The level of experience varies: three operators have worked in the unit for at least 5 years, three others have worked there for between 6 and 10 years, and the remaining two have worked there for over 11 years. Each operator is trained initially for 3 months and then reassessed annually. The mean annual increase in cancer drug compounding is about 4.8 %, and over 52,000 cancer drug formulations were prepared in 2022. The number of clinical trial compounding operations (5700 in 2022) is increasing by about 15 % a year.

### 2.2 Description of the AR eyewear

The AR application was developed as part of a collaboration between the *Groupe de Recherche sur les formes Injectables et les Technologies Associées* research group at the Lille Faculty of Pharmacy (University of Lille and Lille University Hospital, Lille, France) and the *Centre de Recherche en Informatique, Signal et Automatique de Lille* (CRISTAL) research group at the *Ecole Centrale de Lille* (Lille, France). The application was then integrated into Lille University Hospital's ODMS, in collaboration with the latter's developer (Computer Engineering SARL). Two HL7-FHIR connectors were developed, so that the ODMS could be linked to the AR software.

**Fig. 2:**
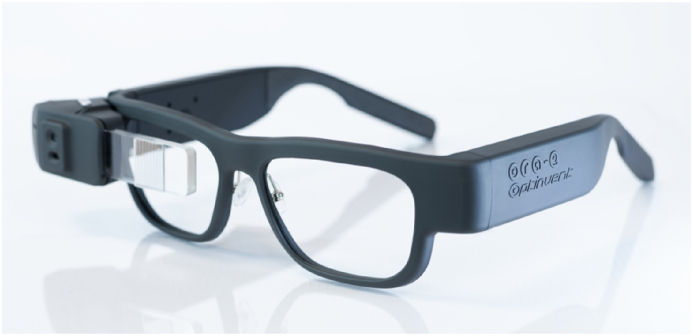
The Ora-2® AR eyewear from Optinvent.

**Fig. 3:**
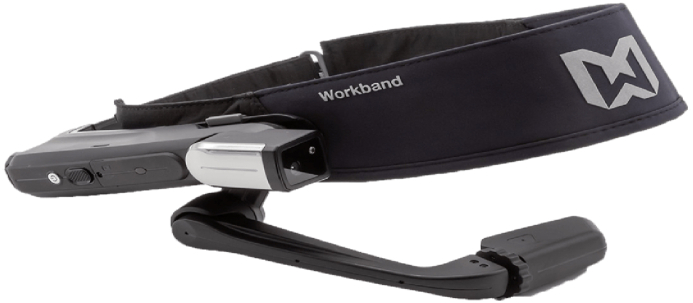
The HMT-1® AR eyewear from RealWear.

Two types of commercially available AR eyewear (the Ora-2® from Optinvent (Rennes, France) (Fig. 2) and the HTM-1® from RearWear, Inc. (Vancouver, WA, USA) (Fig. 3)) were initially tested, in order to select the device that best matched the lab's practices and that gave the best results. The selection criteria were the ability to connect to a secure Wi-Fi network, network integration by the hospital's IT department (given that the device might be a potential entry point for IT attacks), ease of decontamination, robustness versus the chosen decontamination method, and, lastly, wearability and user-friendliness during drug compounding. We finally chose the HMT-1® AR eyewear from RearWear, which comprises a camera on a headband and an adjustable arm that can be placed on the right or left eye (depending on the operator's preference) and even if the operator wears spectacles. Along with better ergonomics for the operator, the HTM-1® device from RealWear offers better screen legibility, higher photo quality, and more sensitive voice recognition. We can only really comment on the model (the HTM-1® from RealWear) that we actually used for the compounding process – we did not perform a comparative study. Several models are currently on the market. One of the most important criteria is the ability to connect to the hospital network while meeting security requirements. This criterion alone reduces the choice considerably. We therefore decided to choose an affordable model that met our security requirements.

### 2.3 The device's operating principle

Each compounding procedure calls on a series of steps preconfigured in the ODMS. These steps can be modified by the hospital, depending on how the preparation will be packaged. Processes are defined for each type of compounding operation in bags, syringes or portable elastomeric infusion pumps, rather than for each drug. Each preconfigured process can then be used regardless of the number of flasks, the pharmaceutical formulation (a solution or a lyophilizate) or the occasional compounding operations involving both unopened and opened flasks.

**Fig. 4:**
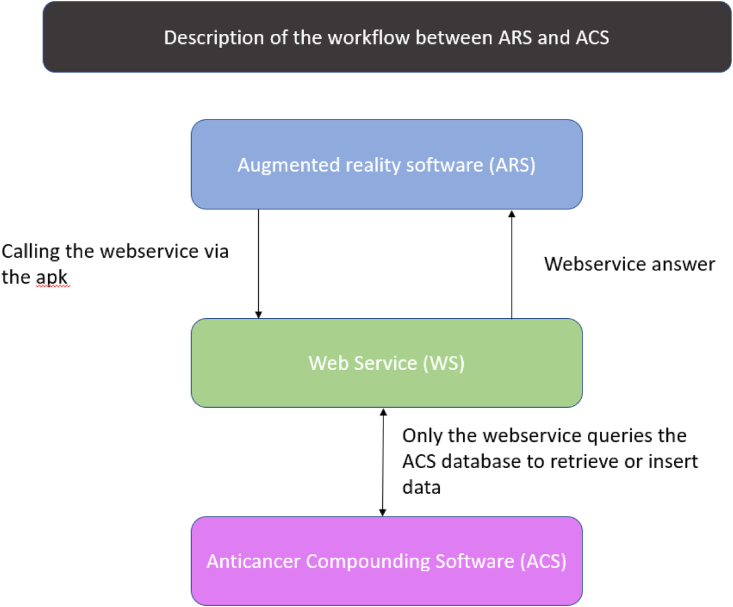
Schematic diagram of the information flow between the ODMS and the AR eyewear.

The eyewear contains onboard AR software that (i) analyzes barcodes, (ii) sends information to the eyewear's screen, in response to voice commands, (iii) takes photos, and (iv) sends information from the eyewear to the ODMS. The AR application (written in Kotlin) is linked to the ODMS via a web service with a representational state transfer architecture. After reading a preparation's specific barcode, the AR application queries the web service. The web service then searches for and recovers the information from the ODMS and sends it to the AR application (Fig. 4). If the information read by the eyewear does not correspond to the information in the ODMS, the AR application displays a warning and blocks further steps in the compounding. After each step has been validated, the web service sends back traceability data and photos of the preparation to the ODMS so that these data can be recorded and linked to the master file.

**Fig. 5:**
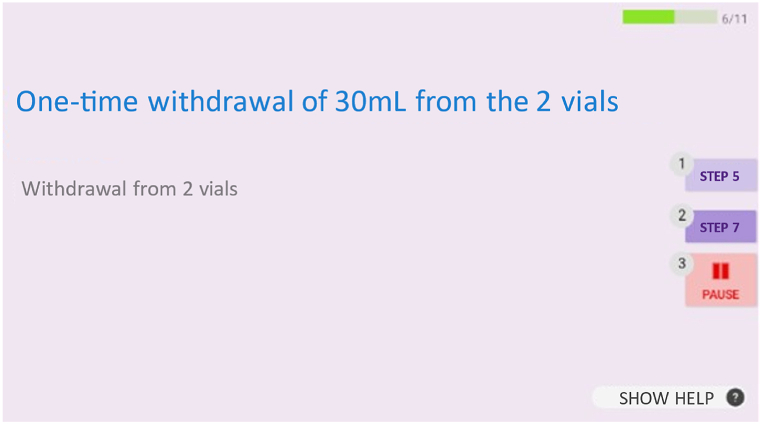
An example of the eyewear's visual display during a compounding procedure.

When the eyewear is being used, the AR application will first ask the operator to identify him/herself by scanning his/her personal Data Matrix code. Once the operator has been identified, he/she can read the code corresponding to the desired compounding procedure; this launches the steps defined in the ODMS via the web service. The available voice commands (e.g. for selecting the next step, changing the zoom level, or taking a photo) are displayed throughout the preparation sequence. The photos must not be blurred and must include the expected elements before they are validated with the voice command “validate”, prior to the next step (Fig. 5).

**Fig. 6:**
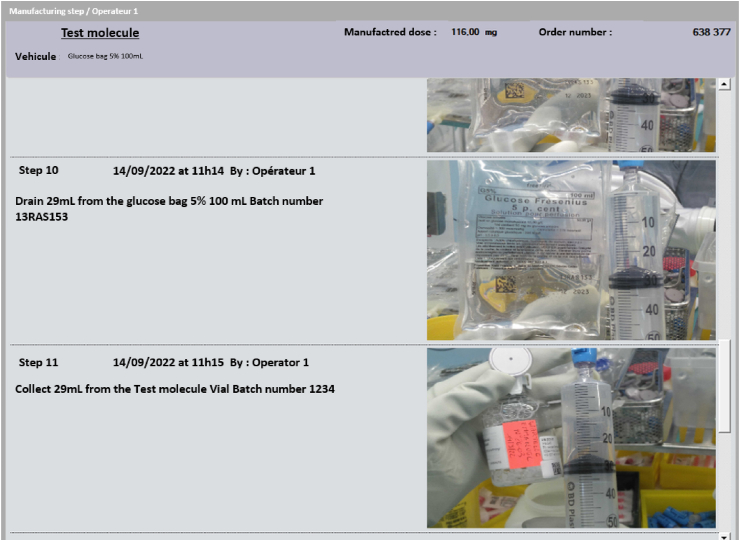
An example of the ODMS's supervision screen for drug compounding with the AR eyewear.

Once the drug has been compounded, all the photos taken and the steps performed can be consulted in the ODMS via the compounding supervision screen, so that the compounded preparation can be released (Fig. 6).

### 2.4 Qualification of the AR eyewear and the staff training plan

The eyewear's design, installation, operation and performance were qualified. The tests' specifications were drafted as Microsoft Word® (Microsoft Corporation, Redmond, WA, USA) documents over a three-week period in January and February 2022. All possible combinations of the following compounding processes (with both unopened and opened flasks) were considered: (i) a bag prepared by reconstitution of a lyophilizate, (ii) a bag prepared from a ready-to-use solution; (iii) a syringe of pure solution prepared by reconstitution of a lyophilizate, (iv) a syringe of pure solution prepared from a ready-to-use solution, (v) a syringe filled with diluent, prepared by reconstitution of a lyophilizate, (vi) a syringe filled with a diluent, prepared from a ready-to-use solution, (vii) a portable elastomeric infusion pump prepared by reconstitution of a lyophilizate, (viii) a portable elastomeric infusion pump prepared from a ready-to-use solution, (ix) an empty bag prepared by reconstitution of a lyophilizate, and (x) an empty bag prepared from a ready-to-use solution.

During tests featuring deliberate errors (i.e. use of the wrong drug or the wrong solvent), the application blocked the compounding process because it was not able to match the recorded data with the expected data.

**Fig. 7:**
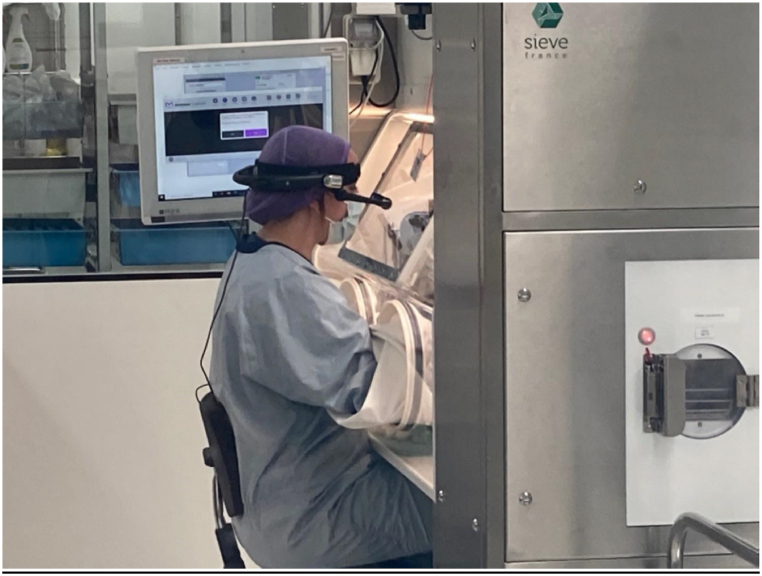
An operator equipped with AR eyewear working on an isolator at Lille University Hospital. The screen is set to one side, so that the trainer can see the information sent to the operator.

Small groups of operators were trained during a 1-h session outside the preparation area. The training was based on an imaginary compounding procedure, which was not shown before the session. The training session was led by an intern pharmacist as part of his PhD thesis, together with one of the pharmacists who designed the application. After this initial training, the operators were allowed to handle material with the eyewear in the drug preparation production area. The first 10 procedures were monitored and double-checked by the trainer, so that he/she could answer any questions the operator might have and resolve any problems encountered. A system for duplicating the eyewear's visual information on a computer screen was set up for the initial compounding procedures, in order to facilitate the training process (Fig. 7).

### 2.5 The study period and the variables recorded

The study was performed from March 1st to August 31st, 2022. The following data were collected from the ODMS: (i) the number of compounding procedures, (ii) the monthly number and proportion of injectable investigational cancer drugs that could potentially be compounded using the AR eyewear, (iii) the monthly number and the proportion of injectable investigational cancer drugs actually compounded using the AR eyewear, (iv) the number of different operators having used the AR eyewear, (v) the number of preparation rejections and noncompliant compounding procedures, (vi) the number and nature of incidents encountered, (vii) the quality of the photos and the number of missing or unusable photos (all analyzed by the same intern pharmacist), in order to determine whether the volume could be read reliably and whether the photo included all the expected information, and (viii) the storage volume required for the photos.

### 2.6 The user satisfaction survey

We surveyed levels of satisfaction among the operators having used the AR eyewear between August 1st and August 31st, 2022 (Appendix 1). The survey was developed on paper, validated by the project's stakeholders, and transposed to Microsoft Forms (Microsoft Corporation, Redmond, WA, USA) to facilitate data collection. The survey was split into five sections (each of which comprised 4 to 12 questions) on (i) the equipment (comfort when worn, screen clarity, and user-friendless in general); (ii) the AR application (use of the application during the preparation, the clarity of the voice commands, the instructions given by the application at each stage in the preparation, etc.); (iii) the eyewear's organisational impact; (iv) the preparation release check (reserved for the pharmacists in charge of releasing preparations), and (v) errors and problems encountered during use of the eyewear.

For closed questions, the operator had to answer on a Likert scale from 1 (“no” or “very dissatisfied”) to 5 (“yes” or “very satisfied”). A “not applicable” answer was also possible. Each section included open questions to which free text answers could be given – notably to collect ideas for improving the system. The survey comprised a total of 42 questions: 8 for pharmacists only and 34 for all users.

## 3 Results

### 3.1 The level of compounding activity

**Fig. 8:**
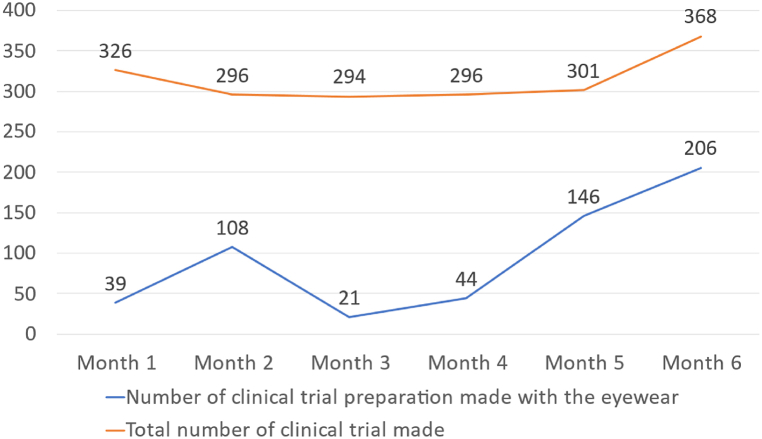
Changes over time in clinical trial drug compounding with the new AR eyewear and in clinical trial drug compounding overall.

During the 6-month study period, 564 investigational drug formulations were compounded by operators equipped with the AR eyewear. The proportion of investigational drug formulations that could be prepared with the AR eyewear ranged from 77 % (251 out of 326 in month 1) to 89 % (263 out of 296 in month 4). The proportion of drug formulations prepared with the AR eyewear increased from 12 % (39 out of 326) in month 1–56 % (206 out of 368) in month 6 (Fig. 8).

Between March 2022 and June 2023, we had produced 2396 clinical trial preparations and stored 15,578 photos.

By month 6, all the unit's operators had been trained and were using the eyewear on a regular basis. Seven operators worked at compounding workstations because one operator was absent during month 6. All the operators used the AR eyewear for much the same length of time (mean ± SD time per preparation per user: 5.7 ± 3.84 min; mean total time over the 6 months: about 7 h per user).

**Fig. 9:**
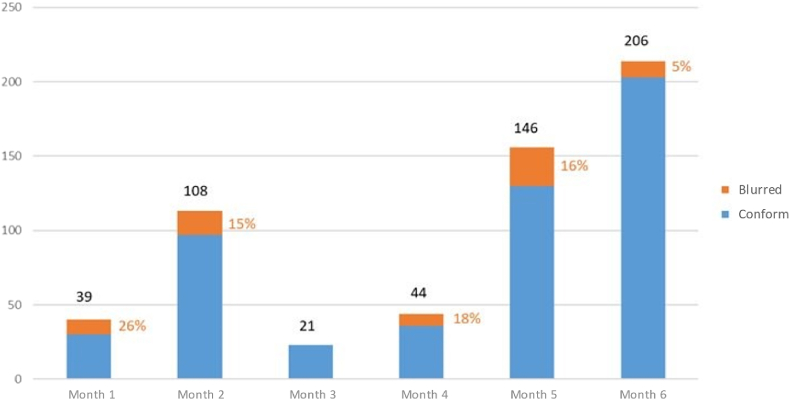
Changes over time in the proportion of blurred photos taken with the AR eyewear.

With regard to the quality of the photos taken with the eyewear, the proportion of poor-quality photos fell from 26 % (10 out of 39) in month 1–5 % (10 out of 206) in month 6 (Fig. 9).

When considering the photos taken for the 564 preparations, we identified three causes of noncompliance: blurriness in 68 (12 %), poor framing or a missing photo in 8 (1.4 %), and operator misuse of the eyewear (noncompliance with the order of the instructions) in 2 (0.3 %).

None of the preparations was rejected during the 6-month study period. Items that could not be properly viewed (due to blurred photos or interface problems) were recovered during the preparation release check (particularly the flasks, if the preparation number could not be read). The photo storage volume required for the routine use of the eyewear (564 compounding procedures) was about 2 Gb in total, i.e. about 3.5 Mb per compounding operation.

Although we did not count the number of incidents, use of the eyewear was affected by various disconnection problems and poor access to the web service during month 3. These problems were resolved by a factory reset of the device and reinstallation of the AR software; routine use of the eyewear was then resumed. Other incidents (related to incorrect use of the eyewear) led to a number of malfunctions, such as unintentional changes in the application's language setting and in the URL used to access the web service. These problems were rapidly resolved, and the AR application was modified (notably through the creation of specific access rights) in order to lock this information and limit access to these parameters.

### 3.2 User satisfaction survey

Twelve people (3 pharmacists, 8 operators, and 1 intern pharmacist, i.e. all the people authorized to use the AR eyewear) completed the survey (Appendix 1).

#### 3.2.1 Evaluation of the equipment

**Fig. 10:**
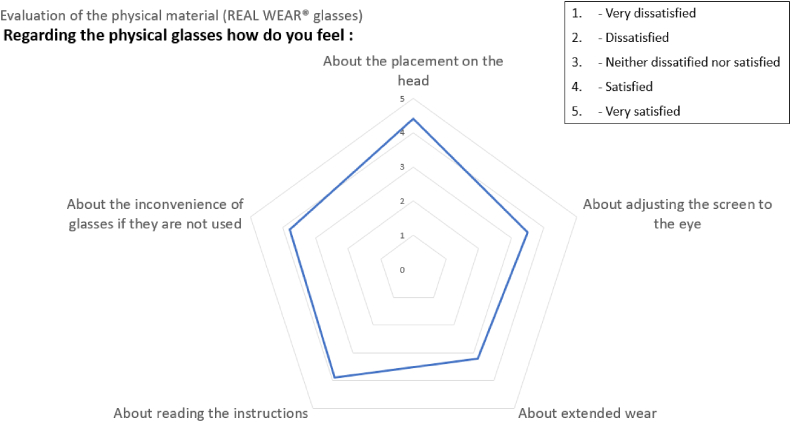
The user satisfaction scores for the evaluation of the equipment.

The radar diagram in Fig. 10 shows the scores for the five questions concerning the selected RealWear® eyewear.

#### 3.2.2 Evaluation of the AR application

**Fig. 11:**
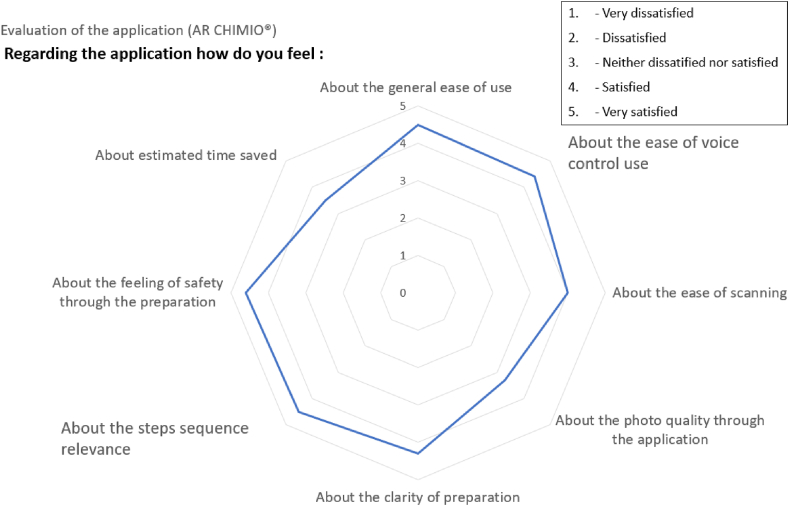
The user satisfaction scores for the evaluation of the AR application.

Fig. 11 shows the scores for the evaluation of the AR application.

Five of the eight operators who had used the eyewear on a routine basis considered that between 1 and 3 compounding procedures were needed in order to feel at ease with the device (Fig. 11). The other three operators considered that 4 to 6 compounding procedures were need. Overall, the mean ± SD number of compounding procedures required was 3 ± 1.6.

The following areas for improvement were suggested: (i) regular training when updates with an impact on use were issued; (ii) training on the resolution of recurrent problems, like unintentional locking of the eyewear by the operator), (iii) an obligation to log in by voice when a user starts to use the eyewear at a workstation, and (iv) a change in the step order for drugs requiring reconstitution in a flask (reading the diluent bag after reconstitution of the flask, to avoid confusion between the diluent and the reconstitution solvent).

#### 3.2.3 Evaluation of the device's impact on operating procedures

**Fig. 12:**
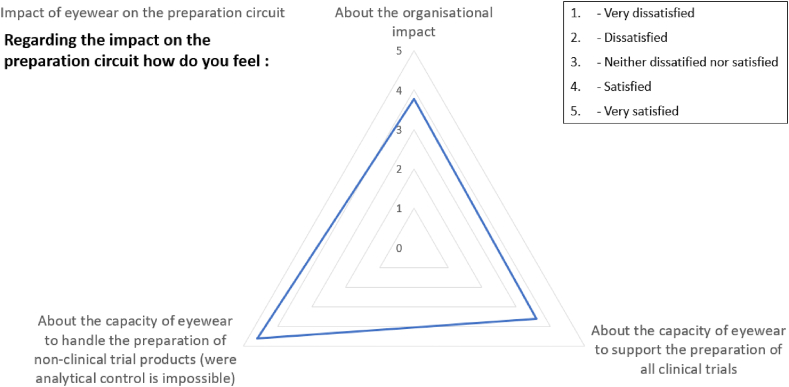
The user satisfaction scores for the eyewear's impact on the investigational drug preparation.

Fig. 12 shows the operators' scores for the device's impact on the unit's operating procedures.

The operators considered that use of the eyewear did not have a negative impact on the drug compounding procedures (Fig. 12). Furthermore, the operators considered that the eyewear could be used to (i) compound all the investigational drugs and (ii) check all the drug compounding procedures. This is an area for improvement envisaged by the development team; use of the AR eyewear for compounding that still currently requires a double visual inspection.

#### 3.2.4 Evaluation of the preparation release check

**Fig. 13:**
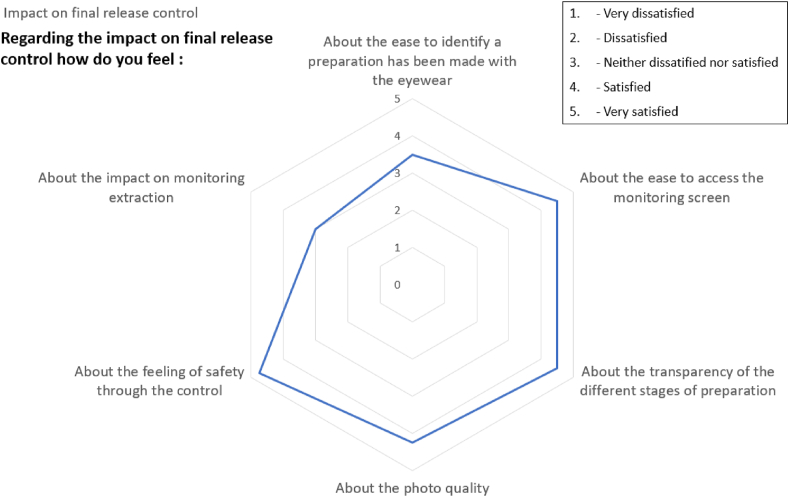
The user satisfaction scores for the impact on the preparation release check.

Only the three pharmacists replied to the six questions about the new tool's impact on the preparation release check (Fig. 13). One suggested improvement for the batch release check was easier access to traceability data and, notably, the ability to zoom in on the photos included in the compounding file. This request has since been taken into account and implemented by the ODMS's developer.

### 3.3 Evaluation of incidents

The most frequently reported problems were network connection failures during the study period. There were no reports of incidents related to the AR eyewear *per se*. The quality of the network connection has since been improved, thanks to work by the hospital's IT department.

## 4 Discussion

The computerized drug compounding process and the integration of innovative support tools preparation and quality control are part of our continuous improvement policy. However, it is sometimes difficult to integrate new technologies because they require changes in compounding and quality control processes. The provision of information, the implementation of qualification procedures and the evaluation of use enabled us to increase the level of safety in the compounding circuit and support our staff during these changes.

### 4.1 Integration of the eyewear into the investigational drug compounding process

We noted eight major advantages of using AR eyewear. Firstly, the device increased safety levels, thanks to (i) traceability of photos that are directly saved in the corresponding compounding file, (ii) fewer interruptions because the double visual inspection now takes place outside the preparation zone, (iii) recording of the steps performed, so that the operator can resume the preparation in the right place after an interruption, and (iv) the display of an error message that prevents the user from moving to the next step when the wrong drug (in a bag or flask) is used. As mentioned above, we consider that the photo storage volume is acceptable, given the corresponding improvement in traceability.

The second advantage relates to the fact that the AR eyewear optimizes an operator's training, due to the step-by-step support provided during the compounding. The eyewear enables the operator to focus fully on the procedure because the step in progress is always displayed in the field of vision. The mental burden is lower because the operator knows that he/she will not forget a step. The benefits of AR for training healthcare professionals are now well documented [10], even though there are few reports on the use of this technology in hospital pharmacies.

The third advantage is a short qualification period, relative to the introduction of an automated production system (e.g. 11 months for the RIVA® compounding robot in our unit (unpublished data)). In comparison, the AR eyewear was implemented and qualified in 4 months. This new tool has integrated well into our pharmacy's compounding activity and is particularly well suited to clinical trials because it is flexible and relatively cheap to purchase.

The fourth advantage is that the AR eyewear only requires an overall configuration for each type of container (a bag, a syringe, etc.), rather than case-by-case configuration for each drug. This process can be adjusted according to a unit's compounding habits. If a unit wants to install this tool, it just needs to draw up a list of types of container (usually fewer than 10) and configure them. In view of the relatively low number of types of container, this configuration is quicker than for a compounding robot.

The fifth advantage stems from the chosen eyewear's user adaptability: the moveable arm can be adjusted to the user for optimum visibility, regardless of the distance between the eyes or the preferred eye. These parameters are optimized during a new user's first compounding operations.

The sixth advantage relates to the short learning curve for optimal use of the eyewear by a new user – estimated at between 3 and 6 weeks, depending on the user's familiarity with IT applications. Given the lack of literature data, we cannot compare this learning curve with those of other devices or methods.

The seventh advantage is wearability. The eyewear is not attached to a workstation and can thus be transferred from one isolator to another; different categories of drug can be compounded without the need to purchase specific equipment. Hence, the eyewear can be deployed in both small units (in order to meet the latter's safety and quality control needs for compounding on a single workstation) and large units (where the eyewear can be used on several workstations). In our unit, the eyewear is used on several workstations, depending on availability. This mobility enables helps to maintain the fluidity of our compounding schedule.

The eighth and last advantage relates to the absence of interference between devices. In fact, the implementation in our unit demonstrated that voice commands for one set did not interfere with those of another set being used at the same time. This is due to the eyewear's directional microphone. It is also noteworthy that the eyewear's data flow is not slowed down by the simultaneous use of a second device because the communication between the AR software and the ODMS via the web service does not affect the Wi-Fi network's data transfer rate.

The currently used version of the AR eyewear also presented several drawbacks. The first drawback was the retrospective quality control of the preparation, using the preparation's master file. If an error cannot be corrected, the preparation must be discarded and the whole compounding process must be repeated. The operator must be careful to comply with the instructions given by the application, such as taking photos - a critical aspect of retrospective quality control. It will be necessary to check the quality of the photos 12 months after the introduction of the device.

Secondly, the operator has to monitor the interface between the AR software and the ODMS. When the device was first introduced (i.e. during the installation qualification phase), occasional Wi-Fi network connection problems prevented the AR eyewear from operating correctly. Hence, during the set-up phase, we worked with our IT department to securely include the AR eyewear in the hospital information system without perturbing the latter.

Thirdly, the AR eyewear was connected to the hospital information system and thus could potentially expose the latter to network attacks. The implementation phase required us to first spend time configuring the device and protecting the network, with the use of a secure Wi-Fi connection. Reports on technical incidents will be analyzed in the future, so that we can gauge the operators’ opinions of the eyewear without being biased by the recent network problems [11].

As the operators were trained in use of the device, we observed a progressive increase in the number of investigational drug compounding operations performed with the eyewear. However, this number fell in month 3, when a network problem prevented use of the system with the eyewear. There are two explanations for the inter-operator differences in time spent using the eyewear immediately after implementation of the system. The first was the series of training sessions for small groups of users, with individual support. The second was the time of year, as the staff went on holiday during the summer.

The greatest change in the compounding process has been the end of the double visual inspection by the operator and a pharmacist; this was greatly appreciated by the unit's staff at a time when human resources were under strain. However, visual inspection is now fully the pharmacist's responsibility, and so an error is still possible. Storage of the photos means that it will be possible to audit the data retrospectively, in order to measure the true error rate for volume measurement in the visual inspection.

AR glasses usefully include information on the preparation steps in the field of vision, and recognize the right components by analyzing the Datamatrix. The main drawback at the moment is inability to determine whether or not the correct volume of liquid has been taken. In our practice (i.e. clinical trial compounding not exceeding 2 h of continuous use), none of the operators have complained about visual fatigue. However, initial use requires the operator to find the right settings.

This study describes feedback from a single preparation team - the first one to use AR glasses. The study's single-center design and the small number of operators are indeed study limitations. We plan to continue this research in a multicenter study and have already identified some interested centres. We can only really comment on the model (the HTM-1® from RealWear) that we actually used for the compounding process – we did not perform a comparative study. More broadly, several models are currently on the market. One of the most important criteria is the ability to connect to the hospital network while meeting security requirements. This criterion alone reduces the choice considerably. We therefore decided to choose an affordable model that met our security requirements.

The glasses replace the bar code scanner - not only because they can scan bar codes but also because they can alert the operator if the product is not what is expected, with an error message appearing in the operator's field of vision. The glasses are more expensive that a bar code scanner but has more functions. From a regulatory standpoint, the software company that developed the data transfer software had to obtain the CE mark. Furthermore, the hospital's information technology department had to check the device's technical characteristics and authorize its use in a care environment.

### 4.2 Perspectives and improvements envisaged

Although the AR eyewear is operating well, it does not yet allow us to comply with all the clinical trial operating procedures. In fact, some trial protocols use specific materials, additional solvents or serial dilutions provided or specified by the trial's sponsor. The device has not yet been configured for these scenarios. One of the first planned improvements will be the ability to configure the system for all clinical trial operating procedures, in order to meet this demand and to fully dispense with the need for conventional double visual inspection. These developments are underway.

Furthermore, a broadening of the eyewear's scope of use would be an interesting option for the compounding of conventional chemotherapies (outside clinical trials) requiring strict quality control (e.g. drug formulations intended for intraspinal administration).

The eyewear could also be used as a training tool for new staff members, thanks to prerecorded training videos that present (for example) the correct techniques during a compounding operation [12,13].

In the longer term, a shift from retrospective quality control to in-process quality control would help to optimize the tool and reinforce the compounding circuit by avoiding the financial consequences of non-compliant preparations.

Lastly, it will be necessary to study the AR eyewear's impact on the compounding error rate. In a preliminary study, we assessed clinical trial compounding based on paper print-outs generated by the ODMS [5]. We counted the number of traceability errors and the number of task interruptions for the staff performing the visual inspection and calculated the time need to complete each step in the clinical trial compounding procedure. Our analysis revealed that (i) the traceability error rate during compounding was 8.8 %, (ii) the inspector responsible for double visual inspection had to check the procedure an average of 1.6 times, and (ii) 51 % of the procedures featured at least one task interruption. In 42 % of the procedures, the inspector had to attend several times, and in 6.7 % of cases, two different inspectors had to check a given preparation. The results of our preliminary study highlighted weaknesses in the compounding circuit – notably with regard to the double visual inspection and disorganization of the work in the production zone, which can lead to errors [5]. The results of the present feasibility study suggest that use of the AR eyewear is likely to increase safety and traceability; however, this question was beyond the scope of the present study.

## 5 Conclusion

Augmented reality eyewear for clinical trial drug compounding is now in operation at Lille University Hospital. This tool has been adopted by the unit's staff and has been integrated into our procedures for compounding investigational drugs; this has fluidified our circuit and made it safer. Although this tool still requires a number of improvements, it opens up the way to higher levels of safety and greater traceability than for double visual inspection. The eyewear is also a key asset when human resources are limited because it enables compounding without task interruptions for the person performing the visual inspection.

The use of the eyewear by other centres could help to broaden the field of use and to check the device's adaptability in other working environments and other organisations. Use of the eyewear could evolve in several ways; this will require collaboration between the various stakeholders, in order to exploit this new tool's full potential.

## Funding

This research did not receive any specific grant from funding agencies in the public, commercial, or not-for-profit sectors.

## Ethics declarations

In accordance with the French legislation on research involving humans, this nonclinical study did not require approval by an investigational review board (article R.1121–1 of the French Public Health Code). Nevertheless, the participants were recruited on a voluntary basis and gave their informed consent to participation.

## Data availability statement

The dataset supporting the conclusions of this article is included within the article.

## CRediT authorship contribution statement

**Arthur Lecoutre:** Writing – original draft, Visualization, Funding acquisition, Formal analysis, Conceptualization. **Michele Vasseur:** Writing – review & editing, Writing – original draft, Project administration, Conceptualization. **Justin Courtin:** Visualization. **Slim Hammadi:** Software, Project administration, Conceptualization. **Bertrand Decaudin:** Validation, Supervision, Project administration, Methodology, Conceptualization. **Odou Pascal:** Validation, Supervision, Project administration, Methodology, Conceptualization.

## Declaration of competing interest

The authors declare that they have no known competing financial interests or personal relationships that could have appeared to influence the work reported in this paper.Michele VASSEUR reports was provided by Lille University Hospital Center. ARTHUR LECOUTRE reports a relationship with Lille University Hospital Center that includes: employment. There is no conflict of interest to disclaim in this article.

## Figures and Tables

**Figure:**